# Integrating Artificial Intelligence and Virtual Reality in Orthopedic Surgery: A Comprehensive Review

**DOI:** 10.1177/15563316251345479

**Published:** 2025-06-17

**Authors:** Robert Koucheki, Johnathan R. Lex, Michael Brock, Danny P. Goel

**Affiliations:** 1 Division of Orthopaedic Surgery, Department of Surgery, Temerty Faculty of Medicine, University of Toronto, Toronto, ON, Canada; 2 Institute of Biomedical Engineering, University of Toronto, Toronto, ON, Canada; 3 PrecisionOS Technology, Vancouver, BC, Canada; 4 Department of Orthopaedics, University of British Columbia, Vancouver, BC, Canada

**Keywords:** artificial intelligence, virtual reality, orthopedic surgery, patient rehabilitation, clinical workflows

## Abstract

Artificial intelligence (AI) and virtual reality (VR) are being used in orthopedic surgery, with goals of enhancing surgical precision, trainee education, patient engagement, and personalized surgical strategies. AI-based predictive modeling, automated computer vision and image analytics, and robotic surgery are changing orthopedic preoperative planning and intraoperative decision-making, with the ultimate aim of improving postoperative outcomes through reduced variability in surgery. VR technologies are being used in orthopedic surgical simulations to provide safe environments for skill development in surgical trainees, helping them practice complex procedures before performing live surgeries. VR platforms are also being studied in-patient rehabilitation, focusing on interactive and gamified approaches that could enhance patients’ adherence, recovery, and outcomes. Major pitfalls and challenges that need to be addressed include technical and logistical barriers, ethical concerns surrounding patient data privacy, and resistance to change among surgeons, trainees, and scientists. Improved infrastructure, standardized protocols, and further research to validate the long-term benefits will be imperative for the integration of AI and VR technologies into clinical and surgical workflows.

## Plain Language Summary

A review of advancements and challenges of integrating AI into the clinical workflow.

## Introduction

Engineering and technological advancements have been used to enhance orthopedic care throughout history. Examples include the use of the Hippocratic ladder for spinal deformity correction (400 BCE), Wilhelm Roentgen’s discovery of X-rays (1895) applied to fracture diagnosis, Sir John Charnley’s pioneering of modern joint replacement implant designs, and Kenji Takagi’s introduction of arthroscopy [42,79,81]. With the rapid technological advancements of the 21st century, the “fifth industrial revolution” has involved innovations such as artificial intelligence (AI) and virtual reality (VR) [69]. Traditional surgical methods are evolving alongside these advancements, with the aim of improved outcomes and enhanced patient experiences. AI and VR have the potential to offer orthopedic surgeons solutions for preoperative planning, intraoperative guidance, and postoperative rehabilitation, but they also introduce new complexities and barriers. This review article explores the integration of AI and VR in orthopedic surgery, their current major applications, benefits, challenges, and future potential.

## Artificial Intelligence

**Fig. 1.**
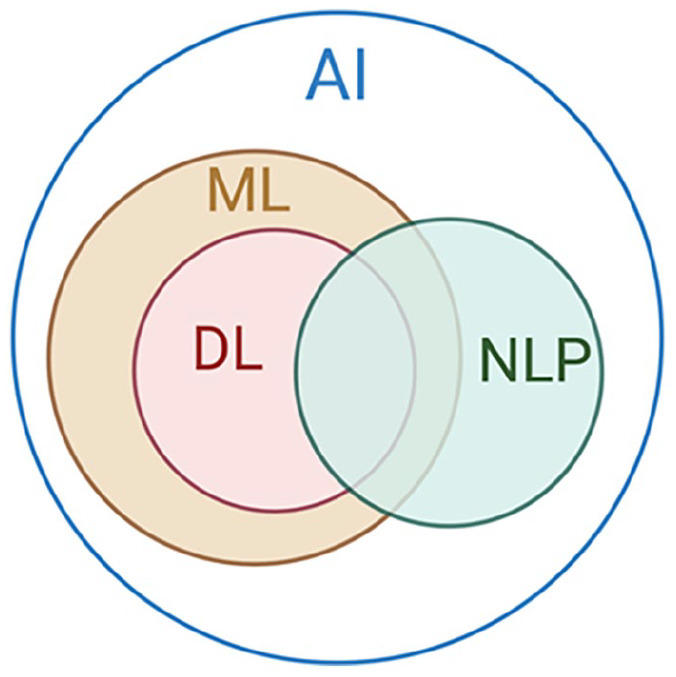
Interrelation between ML, DL, and NLP. *ML* machine learning; *DL* deep learning; *NLP* natural language processing.

AI is coded to use learning, reasoning, and performing self-correction to simulate and automate human intelligence processes by machines [14]. AI is the general term that includes a broad range of technologies, including machine learning, deep learning, and natural language processing (NLP), each of which can contribute to various applications and domains in orthopedic surgery (Figure 1).

Machine learning allows for systems to learn from large datasets to identify complex patterns leading to improved model performance over time. Pattern recognition from large datasets, such as the National Surgical Quality Improvement Program (NSQIP) database or the AAOS American Joint Replacement Registry (AJRR), may be leveraged to lead to improved clinical decision-making. Machine learning can help create predictive models that can optimize patient outcomes through early detection of risk factors for complications, help personalize treatment plans, while possibly improving resource allocation strategies in healthcare settings. Some major machine learning approaches that don’t use deep learning have been used in orthopedic research aiding data analysis including: linear models (such as linear regression, logistic regression, and lasso regression), tree-based methods (such as decision trees, random forest plots, and Gradient Boosting Machines (GBM/XGBoost)), support vector machines (SVM), ensemble learning, K-nearest neighbors (KNN), and unsupervised learning [71].

Deep learning is a powerful subset of machine learning that utilizes deep neural networks to analyze intricate datasets, for example, in medical imaging. Deep learning has been explored in various aspects of orthopedics, including to enhance the accuracy of radiographic interpretation and facilitate three-dimensional (3D) modeling for preoperative planning [28]. Deep learning models have been shown to be highly specific and sensitive in diagnosing and classifying both upper extremity fractures (Table 1) including proximal humerus fractures, elbow fractures, distal radius fractures, and scaphoid fractures and lower extremity fractures including hip fractures, tibial plateau fractures, and pilon fractures [15,21,41,46,48,63,65,86–88,91].

**Table 1.** Major deep learning models in radiographic diagnosis of upper extremity fractures.

Study Author, Year	Joint/Bone	Model Type	AUC / mAP	Sensitivity	Specificity	Notes
Chung et al, 2018 [15]	Shoulder (Proximal Humerus)	Deep CNN	1.00 (fracture detection) 0.90–0.98 (type classification)	0.99 / 0.88–0.97	0.97 / 0.83–0.94	1,891 AP radiographs; outperformed generalists, matched shoulder specialists
Magnéli et al, 2023 [53]	Shoulder (Humerus, Clavicle, Scapula)	CNN	0.89–0.97 depending on bone	–	–	6,172 exams, AO/OTA classification; good generalizability
Rayan et al, 2021 [65]	Elbow (Pediatric)	CNN + RNN (Multiview)	0.95	0.91	0.84	21,456 studies; multiview mimic of radiologists
Ye et al, 2024 [85]	Elbow (Pediatric—Supracondylar)	Multi-scale Patch Residual Network	–	0.89	0.92	GAN-based model; subtle fracture detection; 2-center data
Kekatpure et al, 2024 [32]	Elbow (Distal Humerus—Age ≥7)	ResNet18 CNN	0.787	0.61	0.96	4,931 X-rays; AP & LAT views used
Choi et al, 2019 [13]	Elbow (Pediatric—Supracondylar)	Dual-input CNN (2x ResNet)	0.976–0.992 across test sets	93.9–100%	86.1–92.2%	External validation; comparable to radiologists
Zech et al, 2023 [88]	Wrist (Pediatric—Distal Radius)	Faster R-CNN (Object Detection)	0.92	0.88	0.89	395 PA radiographs; 30% buckle fractures
Raisuddin et al, 2021 [63]	Wrist (Distal Radius—Adult)	CNN (DeepWrist)	0.99 (easy cases)0.84 (CT-confirmed)	–	–	Highlights performance gap in hard cases
Min et al, 2023 [54]	Wrist (Intra/Extra-Articular)	YOLOv5 + EfficientNet-B3 Ensemble	0.82	0.83	0.73	2-stage classification framework
Gan et al, 2024 [22]	Wrist (Distal Radius—Adult)	Unet + Fast-RCNN + DenseNet121	0.941 (ID)0.96 (A/B/C classification)	–	–	2,240 cases; segmentation + classification pipeline
Ju et al, 2023 [31]	Wrist (Pediatric—Trauma)	YOLOv8	mAP@50 = 0.638	–	–	Public dataset; app built for surgical use
Zhang et al, 2023 [90]	Wrist (Distal Radius—Adult)	Deep Learning Ensemble (AP + LAT Views)	–	95.7–98.36%	96.73–98.37%	3,276 AP + 3,260 LAT radiographs; outperformed ortho + radiology attendings
Oh et al, 2023 [59]	Wrist (Distal Radius—Adult)	DenseNet169 + EfficientNet-B0 + HyperColumn + CBAM	0.9145 (with enhancements)0.8778 baseline	–	–	Grad-CAM used for localization; improved performance with attention mechanism
Anttila et al, 2022 [3]	Wrist (Distal Radius—Adult)	Segmentation-based CNN	0.97 (no cast)0.95 (cast)	–	–	Multihospital dataset; pixel-level annotation
Lindsey et al, 2018 [48]	Wrist (Distal Radius—Adult)	DNN + Radiologist-Aided Evaluation	–	80.8% → 91.5% (aided)	87.5% → 93.9% (aided)	135k images; DNN significantly improved ED clinician performance
Rashid et al, 2023 [64]	Wrist (Distal Radius—Adult)	DCNN + LSTM	–	–	–	192 images; Mendeley dataset; class balancing via augmentation
Blüthgen et al, 2020 [6]	Wrist (Distal Radius—Adult)	Deep Learning System (heatmap + classifier)	0.93–0.96 (internal)0.80–0.89 (external)	0.81–0.90 (int)0.64–0.92 (ext)	0.86–1.0 (int)0.60–0.90 (ext)	Performance comparable to radiologists; 524 training images; strong alignment with radiologist ROI
Suzuki et al, 2022 [78]	Wrist (Distal Radius—Adult)	EfficientNet-B2/B4 (Frontal + Lateral CNN)	0.993	98.7	100	Frontal + lateral X-rays; accuracy comparable to hand surgeons
Russe et al, 2024 [67]	Wrist (Distal Radius—Adult)	Classifier, Detector, Segmentor (Comparison)	Up to 0.97 (Classifier)0.94 (Segmentor)	–	–	Head-to-head comparison,; includes commercial tool BoneView
Lee et al, 2024 [44]	Wrist (Distal Radius—Adult)	Attention U-Net + RetinaNet	–	–	–	Measures volar tilt, radial height, inclination; segmentation with high Dice and correlation
Oka et al, 2021 [60]	Wrist (Distal Radius—Adult)	Modified VGG16 CNN (Biplane input)	0.991	98.00%	–	Small dataset; biplanar (AP + LAT) logic improves detection
Suna et al, 2023 [77]	Wrist (Distal Radius—Adult)	2D Dynamic U-Net + Geometric RP Analysis	–	–	–	Automatically calculates 6 radiographic parameters; within expert error margins
Kim et al, 2021 [37]	Wrist (Distal Radius—Adult)	DenseNet-161, ResNet-152	0.962 (DenseNet)0.947 (ResNet)	90.3% / 88.6%	90.3% / 88.4%	Emergency room setting; strong performance with class activation mapping
Gan et al, 2019 [23]	Wrist (Distal Radius—Adult)	CNN + Object Detection	0.96	–	–	2,340 AP images; outperformed radiologists; on par with orthopedists

*AUC* area under the curve; *mAP* mean average precision; *AP* anteroposterior; *GAN* generative adversarial network; *CNN* convolutional neural network; *RNN* recurrent neural network; *DNN* deep neural network; *DCNN* deep convolutional neural network; *LSTM l*ong short-term memory; *CBAM* convolutional block attention module; *YOLO* you only look once.

NLP is a subset of AI that allows machines to interpret, analyze, and interact with human language. In orthopedics, this can be applied for the extraction of clinically relevant data from patient electronic health records including operative reports, consultation notes, and clinical follow-up notes. This capability can help further accelerate and simplify medical documentation, improve clinical workflows, and aid in clinical decision-making.

Each of these components can have applications in different domains of orthopedics including musculoskeletal diagnostics, therapeutic optimization, and operative planning. In musculoskeletal diagnostics, AI-driven image analysis has the potential to rapidly detect pathologies, such as difficult to detect fractures (eg, pediatric or occult fractures), risk factors for osteoarthritis progression, tumor detection, and diagnosis of ligamentous injuries, reducing the reliance on subjective human interpretation. [2,11,46,55,89,92]. Other applications of AI models can predict progression of disease and patient outcomes [17,34]. In preoperative planning, AI can aid surgeons by analyzing patient-specific anatomical variations, to create a more precise, personalized surgical strategy [4,52,70].

AI is also shaping postoperative care and rehabilitation [5]. AI-driven wearable technologies can monitor patient recovery in real time, tracking movement patterns, adherence to rehabilitation protocols, and early signs of postoperative complications such as infections or implant failure [9,72]. Additionally, AI-powered robotic-assisted surgery is enhancing surgical precision, reducing intraoperative variability, and improving patient outcomes in procedures such as total joint arthroplasty and spinal fusion [38].

As AI continues to evolve, its integration into orthopedic surgery holds immense potential to improve diagnostic accuracy, personalize treatment approaches, and optimize both surgical and nonsurgical interventions. However, challenges such as data privacy concerns, algorithmic bias, and the need for rigorous validation remain critical considerations for the widespread adoption of AI in clinical practice [1].

## Virtual Reality

VR is the technology that allows for creation of computer-generated environments, providing users the chance to interact with simulated scenarios in a controlled setting. In orthopedic surgery, VR is being utilized in many areas of clinical practice, including surgical planning, medical training, and patient rehabilitation. By providing realistic 3D environments, VR enables surgeons, trainees, and patients to engage with digital representations of anatomy and procedures, enhancing precision and improving outcomes.

**Fig. 2.**
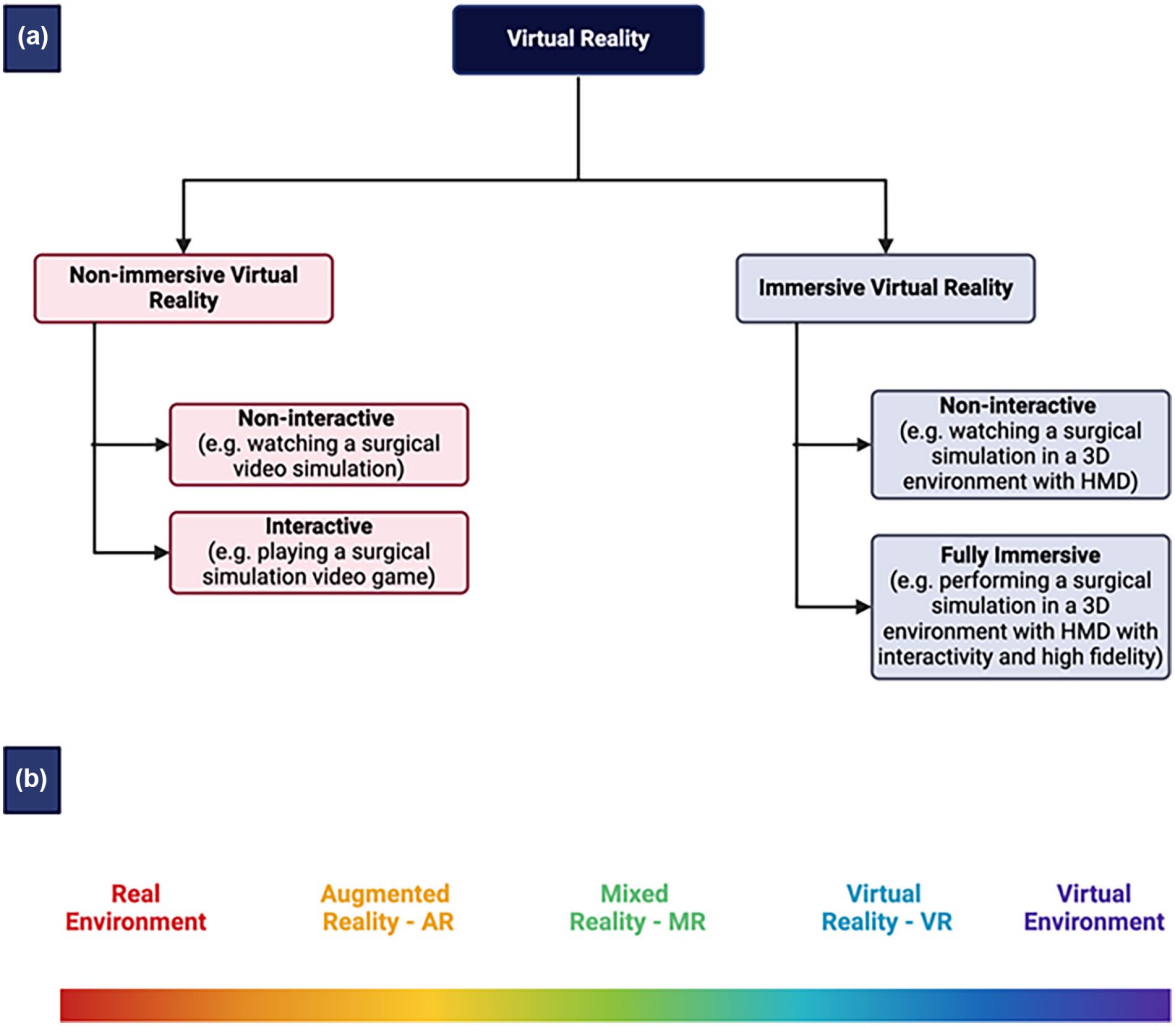
Classification of virtual reality (a). Reality-virtuality spectrum (b). *HMD* head-mounted display.

VR technologies can be classified into immersive and nonimmersive systems, each offering different levels of interaction and integration (Fig. 2a). Immersive VR fully submerges users in a virtual environment using head-mounted displays (HMDs), motion tracking, and haptic feedback, enabling them to manipulate virtual objects as if they were real. This level of immersion is particularly beneficial for surgical simulations and training, where a high degree of realism is needed. In contrast, nonimmersive VR provides virtual experiences through standard computer screens and input devices, offering a more accessible and cost-effective alternative for surgical education and procedural planning (used more in traditional arthroscopy practice).

Related to VR are augmented reality (AR), which blend digital content with the physical world (Fig. 2b). AR overlays virtual images, such as anatomical structures or preoperative scans, onto the real-world environment, allowing surgeons to visualize critical information during procedures without disrupting their workflow [12,62,75,76].

**Fig. 3.**
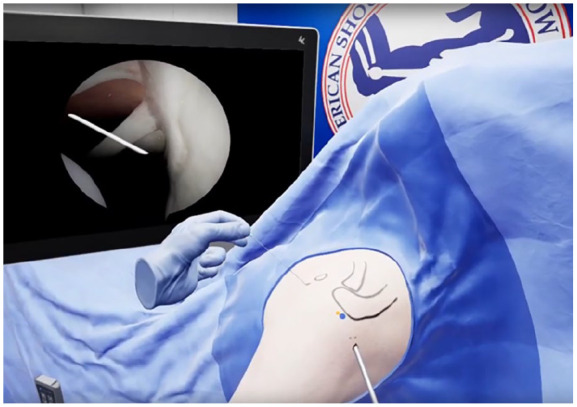
Virtual reality shoulder arthroscopy—permits users to experience and learn principles of arthroscopy in a safe dedicated learning environment (image courtesy of PrecisionOS, Vancouver, BC).

**Fig. 4.**
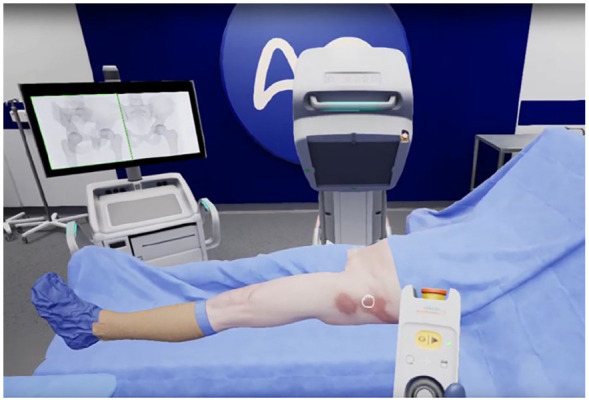
Manipulating a C-arm in VR for simulating and practicing the capture of standard hip projections (image courtesy of PrecisionOS, Vancouver, BC).

VR is currently widely applied in surgical simulations, providing surgeons with a risk-free environment to practice complex procedures before performing them on patients. This hands-on approach allows for skill refinement, improved spatial awareness, and enhanced decision-making in a safe environment. [39,43,50]. Orthopedic trainees can use VR to practice procedures such as arthroscopy (Fig. 3), fracture fixation, and joint replacements, gaining practical experience without the need for cadavers or live surgeries [29,30] (Figure 4).

## Integration of AI and VR in Orthopedic Surgery

To explore the applications of AI and VR in orthopedic surgery, this section examines their role in preoperative planning, intraoperative guidance and robotic assistance, and postoperative care and rehabilitation. Additionally, we highlight their contributions to orthopedic education and training, in particular how surgeons develop technical skills. Finally, we consider their impact on orthopedic research and innovation.

### Preoperative Planning

The integration of AI and VR in preoperative planning has significantly enhanced surgical precision and efficiency in orthopedic surgery. AI-driven machine learning algorithms analyze extensive datasets, including radiographs, CT scans, MRIs, and patient histories to generate detailed, patient-specific anatomical models. These models assist surgeons in developing optimized surgical strategies tailored to individual anatomical variations [57]. AI-powered automated image analysis provides precise assessments of fracture patterns, joint alignment, and soft tissue structures, leading to improved diagnostic accuracy and risk stratification [8,73]. Predictive analytics further enhance surgical decision-making by forecasting potential complications, and allowing for personalized pre-emptive interventions. Additionally, AI-driven implant selection and customization may ensure that prostheses are tailored to the patient’s biomechanics, reducing failure rates and the need for revision surgeries [56].

VR complements AI by providing an immersive, interactive environment for preoperative visualization and planning. VR-based platforms can be used to convert imaging data into high-resolution 3D models, enabling surgeons to explore complex anatomical structures from multiple perspectives. In the future AI-based software may also enable surgeons to use 3D planning that allows trial implant placements, particularly in procedures such as complex fracture fixations (ie, acetabulum and pelvis), revision joint replacements, pediatric orthopedic deformity correction, and spinal deformity corrections. Virtual mock surgeries could enable surgeons and more junior surgical trainees to rehearse procedures in a controlled, risk-free setting, refining their techniques before entering the operating room [51]. Moreover, VR can facilitate collaborative surgical planning, allowing multiple surgeons to interact with and analyze a shared 3D model in real time, improving coordination and decision-making in complex cases [35,80],

### Intraoperative Guidance and Robotic Assistance

AI and VR are playing an increasing role in intraoperative guidance and robotic assistance. AI-driven intraoperative decision support systems provide real-time feedback, assisting surgeons in achieving accurate implant positioning and optimizing alignment during procedures such as total joint arthroplasty, pedicle screw placement, and shoulder surgery [19,40,45,68]. AI-based computer vision systems, enhances intraoperative tissue recognition, enabling surgeons to distinguish between anatomical structures and pathological tissues with greater accuracy [7]. AI algorithms can also process intraoperative imaging and sensor data in real time, providing recommendations that help minimize surgical errors and improve clinical outcomes [49].

VR technology can enhance intraoperative visualization through augmented reality overlays, allowing surgeons to project preoperative imaging data onto the surgical field [16]. This real-time integration can improve anatomical guidance and potentially reduce dependence on traditional imaging techniques. In minimally invasive procedures, VR-assisted visualization could enhance depth perception, making complex techniques such as arthroscopy and endoscopic spine surgery more intuitive and effective. These techniques require hundreds of hours to practice to mastery and application of VR can facilitate this skill acquisition. Additionally, VR enables remote surgical collaboration, allowing expert surgeons to provide real-time guidance and mentorship during procedures, particularly in training settings and resource-limited environments [61,74]. An example of this is a VR training course for emergency and essential surgeries delivered by a UK-Uganda partnership [61]. The combination of AI and VR in robotic-assisted surgery is further advancing precision by automating certain surgical tasks, ensuring consistent outcomes, and reducing intraoperative complications [66].

### Postoperative Care and Rehabilitation

AI and VR developers are trying to transform postoperative rehabilitation by using data-based postop monitoring, for assessment of engagement with therapy programs. AI wearable sensors have the potential to continuously track patient vitals, patterns of motion, and level of adherence to rehab protocols with a potential to detect complications such as infection, implant loosening, and even deep vein thrombosis [33]. Innovative pain sensors and nociceptive management systems could subjectively and objectively assess patients’ pain levels to help adjust medication usage and develop better personalized pain management plans [84]. AI-based predictive analytics can better document individual rate of recovery and may allow clinicians to tailor better rehab plans based on the specific needs of each patient [10].

VR-based rehabilitation programs offer an interactive and engaging approach to physical therapy, enhancing patient motivation and adherence to rehabilitation regimens. Gamified VR therapy transforms traditional exercises into interactive experiences, improving compliance and functional recovery, particularly in patients recovering from ACL reconstruction, total joint replacements, shoulder surgery, or spinal surgeries [24–26,58,82]. VR-based and live motion detectors allow for gait and motion training providing a controlled virtual environment where patients can practice movement, mobility, and balance with real-time feedback [20,36]. Additionally, some VR distraction therapy has been shown to modulate pain perception and reduce anxiety during rehabilitation, improving overall patient satisfaction and recovery experiences [27,47]. By combining AI-driven analytics with VR-assisted therapy, postoperative care is becoming more personalized, efficient, and effective.

### AI and VR in Orthopedic Education and Training

**Fig. 5.**
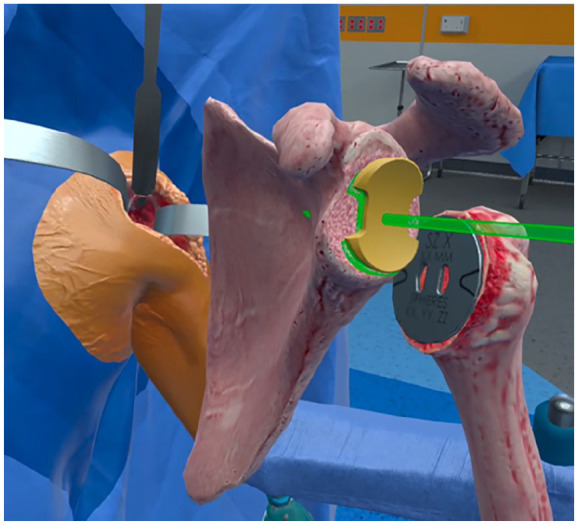
Immediate feedback and visual representations of an ideal positioning within anatomic shoulder arthroplasty enhance the experiential learning value of virtual reality-based training. (Image courtesy of PrecisionOS, Vancouver, BC).

AI and VR are enhancing orthopedic education and training by providing tools that help enhance complex skill acquisition, surgical proficiency, and decision-making (Fig. 5). AI can be used to help personalize educational content based on individual learning patterns of surgical trainees, which could lead to optimizing knowledge retention and technical competence among orthopedic residents and surgeons. Surgical skill assessment systems can use sensors and AI-based algorithms to evaluate procedural motion patterns and accuracy. These platforms can then provide objective feedback that helps trainees refine their techniques and surgical skills. AI can be used for knowledge extraction to help surgeons automate literature reviews, allowing surgeons to stay up to date with the latest research and best practices in the field. AI platforms can potentially update systematic review live as new evidence is published.

VR orthopedic surgery platforms are being used to provide immersive and hands-on experience; these platforms are giving residents the opportunity to practice surgical steps in a risk-free environment. Virtual cadaver labs replicate human anatomy for dissection and procedural practice, reducing reliance on traditional cadaveric training while offering a repeatable, cost-effective alternative [39]. Multiplayer VR surgical simulations facilitate collaborative training, enhancing teamwork and coordination in complex surgical procedures [18,83]. To view a video of an AI agent at work in a virtual environment, go to https://www.youtube.com/watch?v=0d50qXN-fNo.

### AI and VR in Orthopedic Research and Innovation

AI is being utilized in orthopedic research for big data analysis, processing large volumes of patient information to detect meaningful patterns, review treatment efficacy, and optimize clinical guidelines. As mentioned above AI-based systems are currently being studied as literature review tools to help extract and summarize best scientific evidence in a live manner. AI can also be used as for predictive modeling by forecasting appearing trends in musculoskeletal diseases, helping orthopedic surgeon advocate for policies that address emerging healthcare challenges.

3D virtual prototyping of implants, instruments, and surgical devices is another application of VR in orthopedics. Engineers, surgeons, and designers can use VR to design, refine, and test orthopedic instruments or implants in a virtual environment before physical manufacturing, potentially reducing development costs. VR modules are being developed to be used for patient education, providing immersive visualizations that help patient better understand their conditions, steps of surgery, and recovery, and postoperative expectations, helping patient make more informed decisions, and aiding surgeons in the consent process.

## Challenges and Limitations

As with any new technology in medicine, AI and VR pose several challenges and limitations that must be addressed before these technologies can be fully integrated into clinical practice. These barriers include both technical and logistical challenges, as well as ethical concerns related to patient data privacy, resistance to change within the medical community, and the financial burden associated with implementing these advanced tools.

One of the primary challenges that is more easily addressed is the technical and logistical barriers to implementation of AI and VR. These systems require dedicated hardware. Some usages may require robust computing capabilities that will be difficult to integrate with the existing hospital infrastructures. A key concept is that AI algorithms must be extensively trained on diverse datasets to ensure external validity and generalizability across different patient populations and settings. VR surgical training and simulation platforms also require significant infrastructure and computational power with high-resolution displays, which can limit accessibility in resource-constrained environments.

Another important consideration is the ethics of AI/VR, especially the protection of patient data privacy. AI systems rely on patient data for training, validation, and optimization, which raises concerns for data security, informed consent regarding data sharing, and the potential for misuse of personal health information. It is critical that the storage, processing, and sharing of sensitive patient data complies with data protection regulations, such as HIPAA (Health Insurance Portability and Accountability Act) in the United States and GDPR (General Data Protection Regulation) in Europe. It is also important to ensure that AI based decision-making is transparent with explainable logic so that clinical judgments are unbiased and based on sound medical principles rather than opaque algorithms (a risk with unsupervised learning algorithms). Biased algorithms can further reinforce disparities in healthcare and create negative vicious cycles. Resistance to change in clinical practice is another barrier to adoption of these technologies. Many surgeons and trainees may be hesitant due to concerns about reliability, unfamiliarity with these tools, and the perceived disruption of established workflows. Specific solutions include improved infrastructure and investment, robust data, comprehensive security protocols, and education.

## Conclusions

The integration of AI and VR into orthopedic surgery is affecting surgical precision, training for surgeons, patient engagement, and personalized treatment strategies. AI in imaging analysis, predictive models, and robotic surgery is helping to improve preoperative planning, intraoperative guidance, and postoperative recovery. VR simulations are improving and complementing traditional surgical education, giving trainees immersive, risk-free training environments for skill acquisition and improvement. VR-based rehabilitation programs are being developed to improved patient adherence through gamification and recovery experiences, offering more interactive and personalized rehab sessions.

As AI and VR research advances and these technologies become more accessible and utilized in orthopedic practice, they can potentially improve surgical efficiency, safety, and ultimately patient outcomes. While there are still major barriers that need to be addressed, the continuous research and innovation in AI and VR technologies will help redefine orthopedic musculoskeletal care, opening the door to a new era of modern surgical precision, efficient care, and patient-centered surgery.

## Supplemental Material

sj-docx-1-hss-10.1177_15563316251345479 – Supplemental material for Integrating Artificial Intelligence and Virtual Reality in Orthopedic Surgery: A Comprehensive Review Supplemental material, sj-docx-1-hss-10.1177_15563316251345479 for Integrating Artificial Intelligence and Virtual Reality in Orthopedic Surgery: A Comprehensive Review by Robert Koucheki, Johnathan R. Lex, Michael Brock and Danny P. Goel in HSS Journal®

sj-docx-2-hss-10.1177_15563316251345479 – Supplemental material for Integrating Artificial Intelligence and Virtual Reality in Orthopedic Surgery: A Comprehensive Review Supplemental material, sj-docx-2-hss-10.1177_15563316251345479 for Integrating Artificial Intelligence and Virtual Reality in Orthopedic Surgery: A Comprehensive Review by Robert Koucheki, Johnathan R. Lex, Michael Brock and Danny P. Goel in HSS Journal®

sj-docx-3-hss-10.1177_15563316251345479 – Supplemental material for Integrating Artificial Intelligence and Virtual Reality in Orthopedic Surgery: A Comprehensive Review Supplemental material, sj-docx-3-hss-10.1177_15563316251345479 for Integrating Artificial Intelligence and Virtual Reality in Orthopedic Surgery: A Comprehensive Review by Robert Koucheki, Johnathan R. Lex, Michael Brock and Danny P. Goel in HSS Journal®

sj-docx-4-hss-10.1177_15563316251345479 – Supplemental material for Integrating Artificial Intelligence and Virtual Reality in Orthopedic Surgery: A Comprehensive Review Supplemental material, sj-docx-4-hss-10.1177_15563316251345479 for Integrating Artificial Intelligence and Virtual Reality in Orthopedic Surgery: A Comprehensive Review by Robert Koucheki, Johnathan R. Lex, Michael Brock and Danny P. Goel in HSS Journal®
